# Role of Sphingolipids in Multiple Myeloma Progression, Drug Resistance, and Their Potential as Therapeutic Targets

**DOI:** 10.3389/fonc.2022.925807

**Published:** 2022-06-08

**Authors:** Daniela N. Petrusca, Kelvin P. Lee, Deborah L. Galson

**Affiliations:** ^1^ Department of Medicine, Division of Hematology/Oncology, Indiana University School of Medicine, Indianapolis, IN, United States; ^2^ Indiana University Melvin and Bren Simon Comprehensive Cancer Center, Indianapolis, IN, United States; ^3^ Department of Medicine, Division of Hematology/Oncology, University of Pittsburgh Medical Center (UPMC) Hillman Cancer Center, McGowan Institute for Regenerative Medicine, HCC Research Pavilion, University of Pittsburgh, Pittsburgh, PA, United States

**Keywords:** multiple myeloma, sphingolipids, bone marrow microenvironment, drug-resistance, therapy

## Abstract

Multiple myeloma (MM) is an incapacitating hematological malignancy characterized by accumulation of cancerous plasma cells in the bone marrow (BM) and production of an abnormal monoclonal protein (M-protein). The BM microenvironment has a key role in myeloma development by facilitating the growth of the aberrant plasma cells, which eventually interfere with the homeostasis of the bone cells, exacerbating osteolysis and inhibiting osteoblast differentiation. Recent recognition that metabolic reprograming has a major role in tumor growth and adaptation to specific changes in the microenvironmental niche have led to consideration of the role of sphingolipids and the enzymes that control their biosynthesis and degradation as critical mediators of cancer since these bioactive lipids have been directly linked to the control of cell growth, proliferation, and apoptosis, among other cellular functions. In this review, we present the recent progress of the research investigating the biological implications of sphingolipid metabolism alterations in the regulation of myeloma development and its progression from the pre-malignant stage and discuss the roles of sphingolipids in in MM migration and adhesion, survival and proliferation, as well as angiogenesis and invasion. We introduce the current knowledge regarding the role of sphingolipids as mediators of the immune response and drug-resistance in MM and tackle the new developments suggesting the manipulation of the sphingolipid network as a novel therapeutic direction for MM.

## 1 Introduction

Multiple myeloma (MM) is a hematologic malignancy of plasma cells, which is an incapacitating disease characterized by aberrant production of an abnormal antibody called M protein. MM is associated with several other clinical manifestations such as bone destruction, hypercalcemia, anemia, acute renal failure, and reductions in normal gamma globulins. Despite the recent progress in therapeutic approaches, MM currently remains incurable for the overwhelming majority of patients, and the associated bone disease that over 80% of the patients develop constitutes a major source of morbidity and mortality (1, 2). Because of its location in the bone marrow (BM), MM development, from the asymptomatic premalignant stage of monoclonal gammopathy of undefined significance (MGUS) to symptomatic multiple myeloma (MM), is intimately related to its interaction with the cellular components of the BM microenvironment. The genetic instability of the malignant plasma cells induced by oncogenic events and the competition for the occupancy of the supporting BM niche leads to the evolution of clonal tides (3). Furthermore, the BM microenvironment can protect myeloma cells from drug therapies by inducing quiescence, and a subpopulation of myeloma cancer stem cells that post-transcriptionally lose CD138 expression are an extramedullary pool of c-Myc dependent drug-resistant cells (4).

Although cancer research has focused largely on identifying genetic mutations and their expression changes, recent concepts on tumorigenesis transitioned into understanding that metabolic reprograming plays a major role in tumor growth and adaptation. Thus, specific changes in those networks may represent suitable biomarkers and therapeutic approaches. Tumor transformation may cause adaptive alterations within sphingolipid metabolism that cause an increase in pro-survival bioactive sphingolipid species that has pathogenic implications. Sphingolipids are a family of bioactive lipids that have been directly linked to the control of cell growth, proliferation, and apoptosis, among other cellular functions. The association between sphingolipid dysregulation and myeloma pathogenesis is supported by Gaucher disease, a sphingolipidosis characterized by a specific deficiency in an acidic glucocerebrosidase. Gaucher disease patients have a higher risk of developing myeloma and with which share similar disorders including skeletal disease, osteoblast-osteoclast uncoupling, macrophage accumulation, and enhanced osteoclastogenesis (5, 6). New clinical and research developments support the role of BM niche remodeling as a facilitator for tumor transformation. Alterations in both neoplastic cells and cells of the BM niches represent two non-mutually exclusive contributions to myeloma development. Multiple reviews have described the role of sphingolipid metabolic pathways in cancer with an emphasis on ceramide and sphingosine-1-phosphate (S1P) dysregulation (7–12). The role of bioactive sphingolipids in the regulation of malignant hematopoietic cells, in general, and of MM cells is not well characterized.

In this review, we present the recent progress of the research investigating the biological implications of sphingolipid metabolism alterations in the regulation of myeloma development and its progression from the pre-malignant stage, and discuss the roles of sphingolipids in migration and adhesion, survival, and proliferation, as well as angiogenesis and invasion as related to MM. We introduce the current knowledge regarding the role of sphingolipids as mediators of the immune response and drug-resistance in MM and tackle the new developments suggesting the manipulation of the sphingolipid network as a novel therapeutic direction for MM.

## 2 Sphingolipid Metabolism: A Short overview

Sphingolipids are a class of complex bioactive lipids involved in the regulation of almost all cellular functions ranging from the cellular membrane fluidity and barrier function to proliferation, differentiation, adhesion, and cell death (7, 13). The advancements made over the last three decades defined several bioactive sphingolipids as key players in important biologic and pathophysiologic processes and suggested the potential for therapeutic targeting of this pathway.

**Figure 1:**
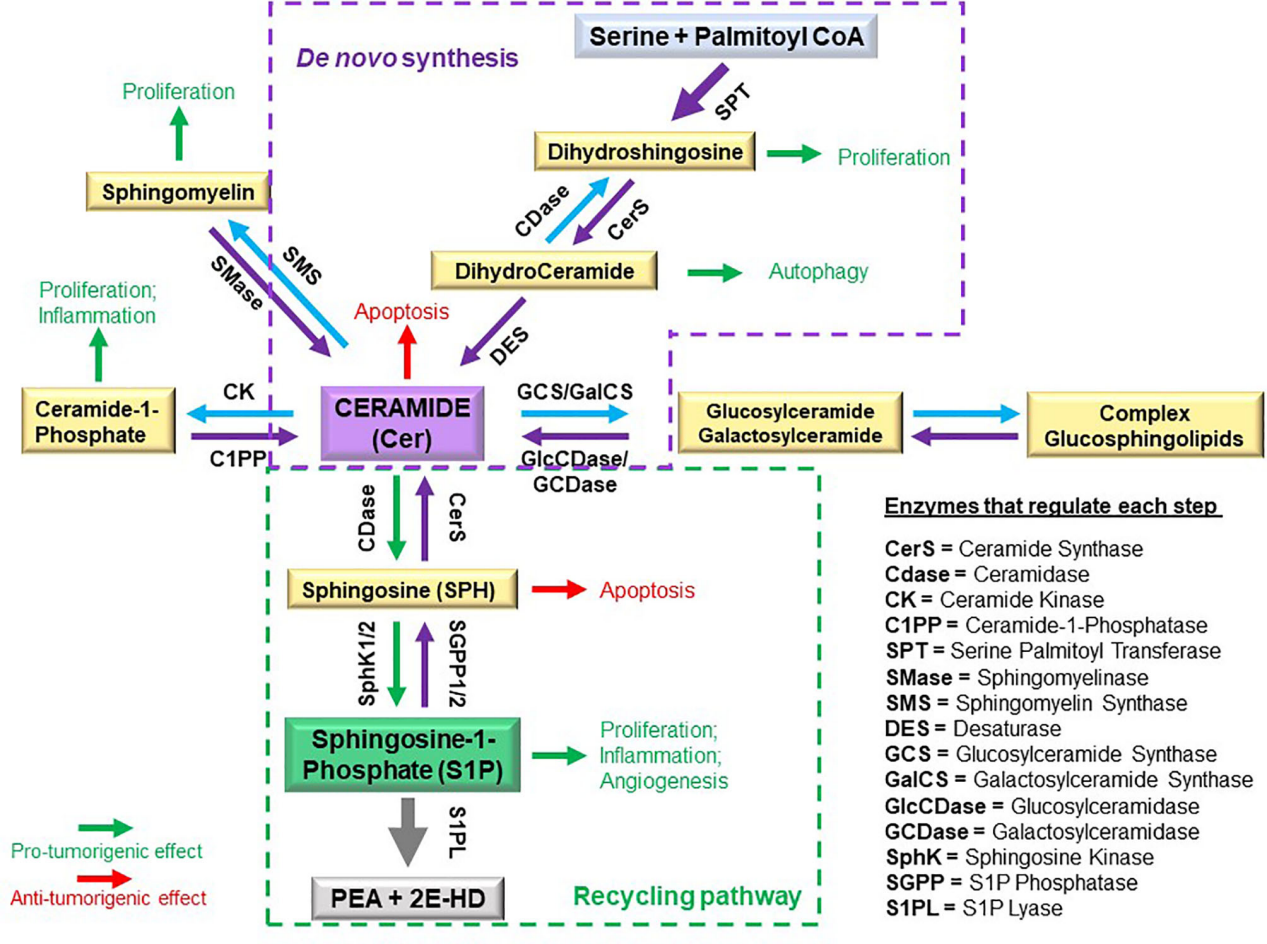
Schematic of sphingolipid metabolism and regulatory interconnections of key enzymes involved in sphingolipid synthesis.

**Figure 2:**
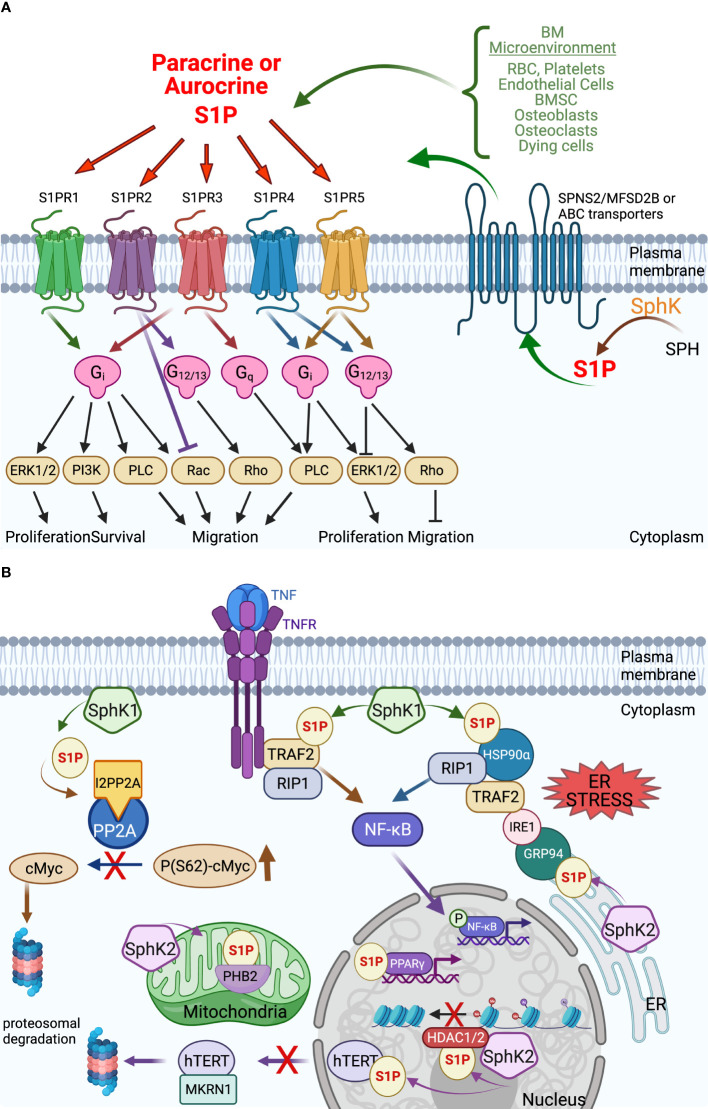
Extracellular and intracellular S1P signaling in the tumor microenvironment. **(A)** Extracellular S1P acts in both autocrine and paracrine by interaction with the 5 S1P G-protein coupled receptors S1PR1-5 to induce an array of downstream signaling with a variety of cellular effects that can vary between cell types. The export of intracellularly produced S1P occurs *via* several transporters from the MFS family (SPNS2, MFSD2B) and the ABC family that also varies by cell type. See text for details. **(B)** Intracellular S1P acts in receptor-independent manners to regulate a variety of proteins – their stability or activation – with effects on cellular functions including gene transcription, chromatin stability, mitochondrial respiration that affect cell viability and longevity. Some examples: S1P generated by HDAC1/2-bound SphK2 binds and inhibits HDAC1/2, thereby opening the chromatin for more gene transcription. The direct interaction of S1P with the transcription factor PPARγ activates it to interact with its gene targets (33). Under ER stress, S1P interacts directly with chaperones HSP90α and ER-localized GRP94 to trigger the formation of a large complex that includes IRE1, TRAF2, and RIP1 to activate NF-κB (34). S1P has also been reported to interact with TRAF2 during TNF signaling to activate NF-κB *via* RIP1 (35). S1P promotes cell viability by indirectly stabilizing cMyc by preventing dephosphorylation by protein phosphatase 2A (PP2A) *via* increasing interaction with the PP2A inhibitor I2PP2A (36, 37). S1P also increases cell longevity by directly interacting with telomerase reverse transcriptase (hTERT) to prevent interaction with MKRN1 and nuclear export to the proteasome (38). Direct interaction of S1P with the mitochondrial protein prohibitin 2 (PHB2) stabilizes it and enhances mitochondrial respiration (39) (created with BioRender.com).

Sphingolipids are amphipathic molecules with hydrophobic as well as hydrophilic properties. The hydrophobic region consists of a sphingoid long chain base to which a fatty acid is attached by an amide bond to carbon 2 (14). While ceramide, the simplest sphingolipid (15), has a minimal hydrophilic region comprised of only two OH groups, the complex sphingolipids contain hydrophilic regions such as phosphate, phosphorylcholine or a sugar (16). Sphingolipid metabolism pathways form a complex network arranged around ceramide, which occupies a central position in sphingolipid biosynthesis and catabolism (14, 17, 18) (**Figure 1** and recently reviewed in (19)). The unique entry point of sphingolipids metabolism is the *de novo* synthesis pathway catalyzed by serine palmitoyltransferase (SPT), which forms 3 keto-dihydrosphingosine (20). Dihydrosphingosine, the reduced form of 3-keto-dihydrosphingosine, can be acylated by a (dihydro)-ceramide synthases (also known as Lass or CerS 1-6) (21) to form dihydroceramide whose desaturation (22) finally leads to ceramide. There is also an alternative pathway, the “salvage pathway”, that leads to ceramide through sphingomyelin (SM) breakdown catalyzed by one of several sphingomyelinases (acid, neutral and alkaline SMases) (14, 16, 20, 23). Ceramide can be subjected to several post-translational modifications as follows: phosphorylation by ceramide kinase (CK) to form ceramide-1-phosphate (13), glycosylation by glucosylceramide synthase (GCS) to form glycosphingolipids (cerebrosides, globosides, gangliosides), conversion to sulfatides by the action of galactosylceramide synthase (GalCS) followed by cerebroside sulfotransferase (CST) (24), or addition of a phosphorylcholine headgroup by sphingomyelin synthase (SMS) to form sphingomyelin (25). Ceramide is also a substrate for several enzymes that break it down and catalyze its downstream products such as ceramidases (CD): acid (ASAH1), neutral (ASAH2) and alkaline (ACER1-3) (26). Activation of ceramidases lead to sphingosine formation, which may be either recycled back into sphingolipid pathways by ceramide synthases (CerS), or it can be phosphorylated by one of the two sphingosine kinases, SphK1 and SphK2, to form sphingosine-1-phosphate (S1P) (27, 28). S1P can act as an intracellular signaling molecule or be exported into the extracellular milieu. Since S1P is amphipathic it requires facilitated export through its specific non-ATP dependent multi-transmembrane transporters Spinster homolog 2 (SPNS2) or major facilitator superfamily transporter 2b (MFSD2B), or *via* several members of the ATP-dependent ABC-type lipid transporters family, namely ABCA1, ABCC1, ABCC2, ABCG2 (12, 29–31), thereby shuttling S1P across the plasma membrane to the extracellular space where it can subsequently act in an autocrine and paracrine fashion (32) (**Figure 2A**). S1P can also either be dephosphorylated to regenerate sphingosine through the action of the S1P phosphatases intracellularly (SGPP1 and SGPP2 (40)) or extracellularly (LLP1-3), or it can be irreversibly cleaved by the S1P lyase to generate ethanolamine phosphate and hexadecenal (41). Although S1P lyase activity represents the unique exit point of the sphingolipid pathway by breaking down S1P into non-sphingolipid molecules, hexadecenal can be reduced to palmitate and subsequently reincorporated into lipid metabolic pathways (41). Intracellularly, S1P acts as a signaling molecule that modulates calcium mobilization (42), mitochondrial respiration *via* interaction with PHB2 (39), proteasomal degradation of TERT (38) and c-Myc (36, 37), and gene expression in multiple ways: *via* direct activation of PPARγ (33), activation of NF-κB *via* direct interaction with TRAF2 (35) or chaperones HSP90 and GPR94 during ER stress (34), and direct inhibition of HDAC1/2 that alters chromatin structure (43) (**Figure 2B**). Extracellular S1P signals through binding to one of five G-protein-coupled receptors (S1PR1-S1PR5) thus participating in regulation of several cell processes such as cell survival, proliferation and migration (**Figure 2A**) (44). The specific effect of S1P is context-dependent being partially determined by the type of the receptors expressed on particular cell types and tumor cells (11).

The best studied bioactive sphingolipids are ceramide and S1P which induce opposite effects in most studied systems, with ceramide inhibiting proliferation and promoting apoptosis (reviewed in (18)) and S1P stimulating growth and inhibiting cell death (45, 46). These observations led to the “sphingolipid rheostat” hypothesis, which proposes that it’s not the absolute levels but the relative amounts of these antagonistic metabolites, S1P and its metabolic precursors ceramide and sphingosine, that determines the cell fate (47). Although widely accepted, the rheostat model has been recently challenged by new studies that found some tumors require ceramide Cer16 for survival or described S1P-mediated apoptotic and autophagic cell death (48–50). Furthermore, recent studies show that ceramide-1-phosphate, a specific ceramide metabolite, is a potent pro-inflammatory mediator, while novel anti-inflammatory roles are being uncovered for ceramide (17, 51–54). Over the recent past, a large variety of studies implicated specific alterations in the metabolic and signaling components responsible for maintaining S1P homeostasis in the development of various cancer types as well as in cell and animal models of cancer. In humans, these molecular changes affect patient prognosis, tumor progression, and resistance to chemotherapy (10).

## 3 BM Microenvironment Cellular Components Release Sphingolipids That Alter the Niche and Support MM Progression

The BM microenvironment is extremely complex consisting of: a cellular component, comprised of hematopoietic cells (hematopoietic stem cells, hematopoietic progenitors, erythrocytes, megakaryocytes/platelets, lymphocytes, monocytes/macrophages, dendritic cells, and osteoclasts) and non-hematopoietic cells (vascular endothelial cells, pericytes, stromal/reticular cells, fibroblasts, osteoblasts, osteocytes, and marrow adipocytes); an extracellular matrix component (fibrous proteins, proteoglycans glycosaminoglycans, SIBLING proteins, adhesion molecules, and the soluble component (cytokines, growth factors and other factors) (55).

In MM, the tumoral cells spread in multiple sites within the BM, and therefore, there is a permanent local interaction with the specialized microenvironment called the BM “niche”, between MM and various BM cells, as well as components of the extracellular matrix. Since the key role of the BM niche in MM development was recognized over two decades ago (56), several experimental approaches were undertaken to determine if either the niche is facilitating malignant transformation of the plasma cells or the malignant plasmacytes transform the normal niche to facilitate their expansion (57). Although the answer to this conundrum requires future work and validation, one thing that remains clear is that the interaction between MM cells and the BM microenvironment niche provides support for MM survival, disease progression and eventually resistance to therapy. Furthermore, the microenvironment, through the release of extracellular signals in the form of cytokines, chemokines, and lipid mediators, works to control immune responses, and inflammation, as well as angiogenesis.

In recent years, sphingolipids became an increasingly studied topic in the field of cancer based on the knowledge that they are essential components of the plasma membrane, modulators of cell–cell interactions and cell recognition and also serve as internal signaling molecules (58). S1P has emerged as a new player in cancer progression that, upon release by different cell types, can regulate the interactions between tumor, immune, and mesenchymal cells present within the tumor microenvironment (11).

S1P is present in the blood, lymph, and interstitial fluid where it is bound to plasma proteins, mainly high-density lipoprotein (HDL) (59), apolipoprotein M (60) and to a lesser extent to albumin (61). However, S1P presence in the BM has been reported (62, 63). S1P can be produced and released into the BM microenvironment by many cell types such as: osteoclast-lineage cells (64, 65), osteoblasts (66), mesenchymal stromal cells (67), and endothelial cells (68). Other important contributors of S1P in the BM are erythrocytes and platelets (69), but also MM cells themselves, as we have recently shown (36). Importantly, S1P can also be released from dying cells (necrotic or apoptotic) and damaged tissues (70, 71), and might create an unwanted pro-metastatic environment as a side effect of chemotherapy (71). This observation, although not yet investigated in the case of the MM microenvironment, might be of high importance since this disease is characterized by increased osteoclasts and impairment of osteoblast progenitors (72).

### 3.1 Sphingolipidomics of the BM and MM Development

#### 3.1.1 Gaucher Disease as a Risk Factor for MM - A Sphingolipid Perspective

Four decades ago, Lee and coworkers suggested a causal link between glucosylceramide storage and occurrence of cancers (73). Over the past three decades, several studies have described an increased risk for MM in Gaucher’s disease patients (the estimated relative risk of myeloma is 5.9 compared to 0.79 in the healthy population (74)), however the reasons for this correlation are currently being debated (6, 75, 76). Gaucher’s disease is a sphingolipidosis with aberrant accumulation of glucosylceramide, primarily within the lysosome, due to a specific deficiency in an acidic glucocerebrosidase (resulting from *GBA1* gene mutations) that hydrolyzes glucosylceramide to ceramide plus glucose (5). The BM microenvironment in Gaucher’s disease seems favorable to plasma cell expansion (6, 77, 78). As a consequence, Gaucher’s disease is associated with an elevated risk of polyclonal and benign monoclonal gammopathy, and it has been suggested that the continuum of disease from either of these two conditions leads to development of malignant gammopathy (5). The connection between dysregulation of lysolipids and myeloma has been reinforced by studies in the context of obesity (79) with the observation that obese persons have a higher risk for myeloma than persons of normal weight (80).

The lack of hydrolysis of glucosylceramide to ceramide in Gaucher’s disease also results in lower ceramide levels. Since ceramide has a well-accepted role in the regulation of cell cycling and the mediation of chemoresistance (81), a decrease in its intracellular levels may predispose to tumorigenesis and impact the response to chemotherapy. The association between elevated glucosylceramide, which accompanies Gaucher’s disease, with the tumorigenesis is supported by several models of cancer (82). First, different studies have shown that neutralization of ceramide to glucosylceramide is a main mechanism of resistance to therapy (51, 83–85). Second, glucosylceramide can block chemotherapeutic-induced NADPH oxidase 2 assembly and subsequent oxidative cell death (86). The most important consideration that associates Gaucher’s disease with malignant transformation is that the glucosylceramide accumulation, or accumulation of complex glycosphingolipids, is intimately linked to the expression of the multidrug efflux pump P-glycoprotein and the development of multidrug-resistance (51, 84, 85, 87, 88). The increased P-glycoprotein expression is likely not carcinogenic in itself, but may exacerbate the progression and severity of malignancies that arise.

Pavlova et al. (89) found that GENZ 112638, a ceramide analog that selectively and potently inhibited GCS, thus preventing production of glucosylceramide and it’s downstream complex glycosphingolipids, when administered to a mouse model of Gaucher disease, ablated the appearance of B-cell malignancies when started early (at 1.5 months), but not when started late (~7 months) after glycosphingolipids had already accumulated in organs and triggered the malignant changes. Further, Nair and colleagues recently showed that glucosylsphingosine mediates B-cell activation in Gaucher’s disease and serves as an antigenic target in Gaucher’s disease–associated monoclonal gammopathy (90). Antigenic reactivity of clonal immunoglobulin has direct implications in understanding the antigenic origins of myeloma and may lead to new strategies to prevent or treat this malignancy by targeting the underlying antigen. Also, it has been suggested that long-term antigenic stimulation may promote genomic instability in myeloma by engaging cytidine deaminases (91). Several studies investigated whether the occurrence of myeloma was a consequence of an inherited error of metabolism since the main manifestation of Gaucher’s disease is the infiltration of the BM by foamy macrophages which are larger than normal macrophages due to accumulated sphingolipids. Macrophages are important cells in the cancer microenvironment and are more prominent in the BM aspirate specimens of MM patients compared with healthy controls (92). Light microscopy analysis of BM aspirate specimens of MGUS (93) and MM patients (94) found foamy macrophages as observed in Gaucher’s disease.

As a result of the complex interactions between components within the MM BM niche, tumor-associated macrophages (TAM) acquire an immunosuppressive and oncogenic phenotype (95). A currently accepted simplified classification, based on the differential polarization that occurs in the tissues, divided TAM into M1 (classically activated) and M2 (alternatively activated). The M1 subtype, triggered by GM-CSF, TNF-α, bacterial products, and interferon-γ, secretes pro-inflammatory molecules, such as interleukin (IL)-1, IL-6, IL-12, IL-23, TNF-α, NO, CXCL9, CXCL10, CXCL11 and reactive oxygen species and may provoke a Th-1 response and play an antineoplastic effect leading to tumor suppression. On the other hand, the M2 subtype is activated by IL-4, IL-10, and IL-13, and expresses anti-inflammatory molecules, such as IL-10, tumor growth factor (TGF-β), CCL17, CCL18, CCL22, mannose receptor C type 1 (CD206), class A scavenger receptor (CD204) and hemoglobin scavenger receptor (CD163), have a low antigen-presenting capacity, and may promote tumor growth and survival by inducing angiogenesis and immunosuppression (96–99). It has been hypothesized that the glycolipid accumulation in Gaucher cells modulates their immune phenotype, leading to increased alternatively activated (M2) macrophages (100).

The prognostic relevance of M2 TAM in MM has been acknowledged by critical clinical studies that associated CD68/CD163 double-positive levels with an increased micro-vessel density and reduced survival, independent of the tumor stage (101–105), as angiogenesis induction is one of the mechanisms through which M2 TAM favors MM progression (104). M2 TAM were found to secrete VEGF and engage in “vasculogenic mimicry” to favor progression from MGUS to MM (106, 107). Furthermore, BM levels of CD206-positive M2 TAM were increased in MM patients with active disease compared to healthy subjects or patients presenting with MGUS (108, 109). In addition, elevated levels of soluble M2 TAM markers CD163 and CD206 were associated with a reduced overall survival, while a higher M1 density was correlated with an overall survival improvement (101, 110).

The propensity of Gaucher’s disease association with increased risk of myeloma has been theorized to result from the accumulation of incompletely metabolized substrates, glucosylceramide, and glucosylsphingosine. Bioactive lipids produced by polarized macrophages have been implicated in mediating the distinct functions of these cells on tissue damage and repair (111). Recently, untargeted lipidomic analysis conducted in mouse BM-derived macrophages (BMDM) identified signature lipid species relevant to the M1/M2 polarization. While M1 BMDMs expressed a ceramide-generation metabolic pattern, leading to elevation of ceramide, M2 BMDMs expressed a ceramide-breakdown-metabolic pattern, resulting in upregulation of S1P (112).

Although additional studies are required to elucidate the mechanistic link, these studies suggest that sphingolipids play a significant role in TAM polarization towards the M2 status, which favors a supportive BM microenvironment for MM homing and proliferation, and thus priming the niche for MM evasion and progression.

#### 3.1.2 Sphingolipid Metabolic Changes in MGUS Condition as a Prerequisite of MM Development

The clonal cell disorder MM is always preceded by the asymptomatic, precursor phase known as MGUS (113). The genomic architecture of MGUS is remarkably similar with that associated with MM, therefore the driver for the malignant transformation may come from the large number of potential players within the BM niche. It is widely accepted that progression of clonal plasma cells, from an indolent MGUS phase to the malignant MM phase, is driven by several oncogenic initiators such as Myc structural variants, activating mutations of RAS and NF-κB pathways (114). Since most oncogenic drivers have downstream effects on various intracellular metabolic pathways (115), cancer cells evolve to adopt a distinct metabolic phenotype that reflects their adaptation to the increased rates of cellular proliferation (116). Therefore, the dysregulation of cellular metabolism is considered a hallmark of malignant transformation and, as anticipated altered cellular metabolism also plays a role in the pathogenesis of MM (117). Based on plasma and intracellular metabolite profile analysis, metabolomics can discriminate between different cellular metabolic phenotypes. So far only a few studies have determined various relative differences of nucleotides, lipids, and amino acids metabolite profiles in the peripheral blood serum or plasma and/or BM plasma between controls, MGUS and MM patients (117–120). A significant observation emerged from these studies pointing out that the detected metabolite alterations vary more between healthy controls and MM than between patients with MGUS and MM suggesting that there is an early metabolic transformation of the BM microenvironment that may not be dependent on significant plasma cell occupancy (117). Quantitative lipid profiling comparing BM plasma from MGUS with MM patients followed by random forest analysis on the differences in the lipid profiles depicted sphingolipids among the top 30 complex lipid species contributing to the group separation (121).

Presently, there is a very limited body of knowledge about the alterations in the levels of sphingolipid species in the blood and BM plasma of MM patients. All these studies report data collected from small patient cohorts and they were mostly designed to determine the methodological feasibility and develop a suitable workflow for future in depth investigations.

### 3.2 BM Niche Factors That Contribute to MM Development by Engaging Their Sphingolipid Metabolism

Metabolic changes are now considered a major characteristic of malignant cells. Loss of a normal microenvironment creates permissive conditions for tumorigenesis and the extracellular stimulation responsible for tumor transformation induces metabolic changes that deregulate the ratio between the various sphingolipid species, their subcellular localization and downstream signaling. Therefore, the intracellular increase of sphingolipid species (which may be released into the microenvironment) in any cell present in the BM niche, including polarized macrophages, osteoblast, osteoclast and MM cells represents a possible source of the deregulated extracellular levels of these sphingolipids. Intracellular ceramide and S1P have been demonstrated to be secreted extracellularly through lipid vesicles/exosomes (122, 123) or transporters (124, 125) (**Figure 2A**). S1P has been implicated in tumorigenesis and its roles in all major hallmarks of cancer were described and extensively reviewed (126, 127). This bioactive molecule, when extracellular, activates a family of G-protein-coupled S1P receptors that couple to various cellular signaling pathways (**Figure 2A**), whereas intracellular S1P directly binds to intracellular protein targets (**Figure 2B**). Since S1P is amphipathic, extracellular S1P has to either be directly generated in extracellular compartments or transported outside the cells by specific transporters after its intracellular synthesis. Sphingosine kinase, the enzyme that catalyzes S1P generation, has two isoforms with distinct biological roles: SphK1 is localized predominantly in the cytosol close to the cell membrane (128) and SphK2 is localized mainly in the nuclear membrane although, depending on the cell type, may also be found in the ER and mitochondria (129). These enzymes can be modulated by a diversity of microenvironmental factors. The proliferation and survival of MM cells are regulated by the BM microenvironment, which also consists of cytokines released from stromal cells and osteoclasts. Activating phosphorylation of SphK1 on Ser225 is thought to link growth factor signaling and bioactive sphingolipid production. Several signaling pathways, such as mitogen-activated protein kinase (MAPK), protein kinase C and PI3K, have been reported to mediate SphK1 activation after stimulation by agonist binding (130). A large variety of factors that can stimulate SphK1 has been described: platelet-derived growth factor (PDGF) (131), vascular endothelial growth factor (VEGF) (132), epidermal growth factor (EGF) (133), hepatocyte growth factor (134), cytokines (tumor necrosis factor-α (128, 135, 136) and IL-6 (137)), steroid hormone (estradiol) (138), and GPCR ligands (acetylcholine) (139), hypoxia (140), S1P itself (141), and many other factors (reviewed in (142–144). Upon activation, SphK1 translocates from the cytoplasm to the plasma membrane and this is an indication of poor MM prognosis. Li et al. demonstrated that IL-6 induces cellular SphK1 activity in MM cells and that this activation mediates the suppressive effect of IL-6 on MM cell apoptosis (137). Since there are no identified activating mutations of SphK1, it has been suggested that tumors become dependent on SphK1 overexpression and/or increased activity which may induce a non-oncogene addiction (145). Our group recently showed that IL-6 and adhesive interactions with stromal cells simultaneously increased SphK1 and c-Myc mRNA and protein levels in several MM cell lines (36).

Although less studied, SphK2 activity can also be further enhanced by phosphorylation events. It has been suggested that ERK1/2 phosphorylates SphK2 on either or both Ser351 and Thr578 (146). In the nucleus, SphK2 interacts with the histone deacetylases (HDAC)1/2 and the locally produced S1P binds to and inhibits HDAC1/2 resulting in increased acetylation of histones H3, H4 and H2B with the consequent increase in the expression of cell cycle related genes (43).

It has been reported that the ceramide pathway is involved in the biogenesis and secretion of exosomes (123). Furthermore, extracellular vesicles originated from MM cells act as essential mediators of short- and long-range communication within the BM (147, 148). Recent studies demonstrated that MM cells exposed to exogenous short-chain ceramides (Cer6) increase both the secretion of exosomes and the exosomal levels of some tumor-suppressive miRs (miR-29b, miR-202, and miR-15a/16), which could target and suppress neighboring MM cells (122).

In the attempt to assess a possible link between sphingolipid metabolism enzymes and the pathogenesis of MM, Watek and collaborators evaluated activity of acid sphingomyelinase (ASM), beta galactosidase, and beta glucosidase using a dry blood spot test in patients with different stages of MM disease. The study analyzed blood samples from 14 newly diagnosed MM patients, 55 MM patients who were on treatment, and 17 healthy volunteers. The results showed that ASM activity, which catalyzes the breakdown of sphingomyelin into ceramide, in the blood of patients diagnosed with MM is significantly reduced compared to healthy donors regardless of the stage of disease. These data suggest a possible role for ASM in MM development. Although these results require further analysis and better understanding of their meaning in the context of MM pathogenesis, they point out a possible diagnostic value for ASM levels (149).

## 4 Sphingolipid Role in the Hallmarks Associated With MM Progression

The function of sphingolipids in tumorigenesis is complex and it depends on cell type, receptor expression, subcellular localization, and environmental context. The discovery that sphingosine inhibits protein kinase C (150) tremendously stimulated research on the roles that sphingolipids might play in regulating various cellular functions including cell proliferation, differentiation, apoptosis, cell survival and death, autophagy, and immune response. Consequently, the sphingolipid research in hematological malignancies provides increasing evidence of the role that the imbalanced “sphingolipid rheostat” contributes to pathogenesis and resistance to therapy. Bioactive sphingolipids have emerged as key regulators of most of the significant attributes of cancer cells. S1P has been implicated in tumor cell survival, cancer cell invasion, cancer inflammatory pathways, angiogenesis, and resistance to chemotherapy. Conversely, ceramides have been implicated in cancer cell apoptosis, growth arrest, mediating sensitivity to chemotherapeutics and senescence (58, 151).

While there is a big body of work regarding S1P signaling in cancer in general, and recent progress has been made in investigating the biological implications of sphingolipids in the regulation of hematological malignant cells, there are only a few studies investigating the roles of S1P and other sphingolipids in the tumorigenic and pathological progress of MM.

### 4.1 Migration and Adhesion

It has been reported that S1P regulates hematopoietic progenitor and plasma cell localization in the BM (152, 153). Myeloma cell trafficking to the BM is mediated by the integrins α4β1 and α5β1 and chemokine–receptor axis CXCL12–CXCR4 (154, 155). α4β1-dependent MM cell adhesion is up-regulated by the chemokine CXCL12 and is crucial for the progression of the disease (156). Elegant studies by Garcia-Bernal demonstrated the involvement of S1P in MM cell adhesion and migration *in vitro* and *in vivo* (63). The authors of this study found that S1P, secreted by MM-exposed BM stromal cells, enhanced CXCL12-promoted MM cell chemotaxis, as well as chemokine-triggered cell adhesion mediated by α4β1 and α5β1. S1PR1 depleted myeloma cells retained full CXCL12 responsiveness while CXCR4 depleted myeloma cells were unable to induce CXCL12-stimulated cell adhesion or transendothelial migration after S1P stimulation. Since both S1P and CXCL12 activate DOCK2–Rac1 signaling that controls α4β1 affinity, the authors suggest that S1P amplifies CXCL12-activated myeloma cell adhesion by further stimulating or maintaining high-affinity α4β1 conformations at early cell attachment steps. Moreover, S1PR1 present on MM cells was found to mediate the optimal interaction of MM cells with the BM microvasculature *in vivo*, thus contributing to their lodgment inside the BM niche. Allende et al. showed that B cell release from BM into blood is promoted by S1PR1 when CXCR4 signaling is weakened (157). Based on this knowledge and their own discoveries, Garcia-Bernal and colleagues proposed that S1P gradients from blood to BM niches with low CXCL12 may constitute a mechanism facilitating myeloma cell egress from BM for metastasis to other tissues.

### 4.2 Survival and Proliferation

As mentioned above, S1P effects on a given cell can be receptor-mediated (autocrine, paracrine and signal amplification loops) and intracellular target protein mediated. Our group showed that exogenous S1P rapidly increases c-Myc protein levels in MM cells, the major contributor to the MM malignant phenotype (36). Extracellular S1P effects are mediated by binding to one or more of the five G protein-coupled S1P receptors (S1PRs): S1PR1/EDG-1, S1PR2/EDG-5, S1PR3/EDG-3, S1PR4/EDG-6, and S1PR5/EDG-8 (**Figure 2A**). G proteins, grouped into four families: Gi, Gq, Gs, and G12 mediate the S1PR downstream signaling pathways including Ras/MEK/ERK, Rac/PAK, and Rho/ROCK (158–160). A few studies showed that S1PR signaling is critical for MM survival. Li and colleagues examined the mRNA expression patterns of S1PR in primary MM and MM cell lines, and reported S1PR1-3, but not S1PR4- 5, are expressed in primary MM, XG-7, SKO-007, and RPMI-8226 cells (161). These authors show that S1P induces the rapid phosphorylation of MAPK, STAT3, and AKT in XG-7 cells and a consequent dose and time dependent upregulation of Mcl-1 protein. Further, using a Gi-protein uncoupling agent, pertussis toxin, and specific pharmacological inhibitors on XG-7 MM cells, they showed that S1P activation of S1PR2 was required for MAPK and AKT signaling, and S1PR3 for STAT3 signaling, and both were involved in the S1P-increased Mcl-1 expression. A clear S1PR1 role was not found. Interestingly, extracellular S1P had a protective effect against dexamethasone-induced apoptosis, and this suggested that S1P levels in the blood stream of MM patients may exert a potential protective effect against this known anti-myeloma drug.

Further, Fu and colleagues reported that S1P activation of S1PRs signaling increased cell proliferation in the U266 MM cell line and three newly diagnosed, never treated, MM patients (162). This was confirmed by significant dose-dependent suppression of viability of both U266 cells and MM patient cells after pharmacological S1PR inhibition with the sphingosine analogue FTY720. Unlike other MM cell lines examined, the authors found that U266 cells expressed the mRNAs of all 5 S1PRs. They focused on S1PR5 and showed that it signals *via* Gi to both Rac1/PAK3 and Cdc42/PAK3 in U266 cells resulting in activation of the PI3K/Akt/mTOR and STAT3/bcl-2 pathways involved in cell survival and autophagy.

Although there is a consensus about the pro-survival effect of S1P stimulation of S1PRs in MM cells, these studies are in disagreement with respect to which S1PRs are present on the surface of MM cells and their roles. As noted above, Li et al. suggested only S1PR2 and S1PR3 are involved in MM cell survival and proliferation, while Fu suggested that S1PR5 is involved in proliferation and S1PR1-4 in drug resistance of MM cells. Moreover, Li reported that U266 cells only express S1PR3, whereas Fu found all 5 S1PR mRNAs were expressed. Since these studies left a series of unanswered questions, the specific role of the S1P receptors in MM functions need further investigation. Furthermore, future receptor studies will need cell line authentication, thorough knock-down driven research for individual receptor types, as well as confirmation of the results in MM patient CD138^+^ cells.

Several sources point to an intracellular role for S1P by binding and altering the function of several intracellular proteins. Recently, our group identified extracellular S1P as one of the soluble factors that provides MM with supportive signals from the microenvironment by increasing Growth Factor Independence 1 (GFI1) transcription factor levels. We found that higher GFI1 levels increase MM cell growth and viability and enhance MM cell resistance to bortezomib-induced cell death *in vitro*, and also increases MM cell growth and MM-induced osteoclastogenesis *in vivo* (163). Importantly, in our most recent study (36), we demonstrated that GFI1 modulates sphingolipid metabolism in MM cells to increase intracellular S1P by acting as a direct repressor of S1P phosphatase 1 (*SGPP1*) gene transcription in all types of MM cells regardless of their p53 status. As a consequence, the S1P:Cer intracellular ratio is tilted toward S1P, thereby supporting a pro-survival, pro-proliferative cellular status. S1P transporter SPNS2 knockdown increased MM viability, confirming that high intracellular S1P is key. The high intracellular S1P level inactivates PP2A through prevention of ceramide competitive interference with the binding of the I2PP2A inhibitor, and thus stabilizes c-Myc expression in MM cells to increase their survival (**Figure 2B**). Moreover, the GFI1 overexpressing MM cells are also releasing a significantly higher S1P amount into the extracellular medium suggesting an S1P-dependent feed-forward loop supporting the MM cells (36). By analyzing the gene expression arrays from the publicly available myeloma GSE6477 microarray dataset, we found progressive and significant repression of *SGPP1* expression with different stages of the disease compared to normal plasma cells, and the most profound inhibition of *SGPP1* expression was found in relapsed MM patients suggesting that changes in sphingolipid metabolism occur during, and may contribute to, disease progression, and therefore may be clinically significant in MM.

Recent evidence from Venkata et al. highlights SphK2 overexpression in MM cell lines and in primary human BM CD138^+^ myeloma cells and its contribution to the survival and proliferation of these cells (164). By also analyzing the publicly available myeloma GSE6477 microarray dataset, the authors show a significant increase in *SphK2* mRNA expression in CD138+ cells from newly diagnosed MM patients as compared to MGUS, while finding no significant difference in SphK1 expression levels. Mass spectrometry analysis of different sphingolipid species showed significant differences only in sphingosine levels, which were lower in 4 of 6 the MM cell lines as compared to two B cell lines, proving consistent with the increased *SphK2* gene expression. This study demonstrated an important role for SphK2 in myeloma pathogenesis since its knockdown inhibited myeloma cell proliferation and induced caspase-3 mediated apoptosis by upregulating Noxa expression and promoting Mcl-1 and c-Myc proteasomal degradation regardless if stromal cells were present. Moreover, specific pharmacological inhibition of SphK2 inhibited myeloma tumor growth *in vivo* in a mouse xenograft model using the MM.1S human myeloma cell line injected *via* tail vein or subcutaneously into sublethally irradiated NSG mice (164).

PIWI-interacting RNAs (piRNA), are non-coding RNAs (ncRNAs) with a length of 24–32 nucleotides and 2’-O-methylation at their 3’-end, which can play both an agonistic and antagonistic role in the development of cancer (165). A recent study showed that S1P-S1PR signaling regulated MM survival through upregulation of piR-004800 (166). After performing small non-coding RNA sequencing on BM exosomes from MM patients and healthy donors, the authors found piR-004800 expressed at a higher level in MM compared to control samples and the levels positively correlated with increased ISS stages of MM. Further, they showed that S1P stimulation of S1PRs increased piR-004800 expression in 2 MM cell lines (RPMI8226 and U266), and that down-regulation of piR-004800 decreased total AKT and mTOR and induced apoptosis and autophagic cell death (166). Although the exact mechanism of how piR-004800 upregulates PI3K/AKT/mTOR (part of the downstream signaling by SIPRs) needs further investigation, this study is the first to report a link between sphingolipid signaling and piRNA regulation to promote MM pathogenesis.

Studies of Gaucher disease indicated a role for glycosphingolipids in the pathogenesis of MM. Ersek and coworkers identified ganglioside GM3 as the most abundant polar glycosphingolipid expressed on myeloma cells, which also release it into the tumor microenvironment (167). Treatment of the preclinical 5TGM1-EGFP murine myeloma model with glycosphingolipid inhibitors induced a reduction in tumor growth and load, thus demonstrating a role for glycosphingolipids on myeloma progression (167).

### 4.3 Angiogenesis and Invasion

A constant characteristic of MM progression is increased angiogenesis, the BM neovessel formation which forms the “vascular niche”, that is critical for its growth, invasion, and metastasis (168). Progression of MM from MGUS is accompanied by significant increase in the BM microvascular density, which is seen also in active versus non-active myeloma (169). The imbalance of pro- and anti-angiogenic factors in the tumor microenvironment promotes the transition from an avascular to a vascular phase of tumor growth, a so-called ‘‘angiogenic switch’’. Through intercellular interaction and the expression of angiogenic molecules such as VEGF, HGF and bFGF, MM cells promote vessel formation (170). The SphK1–S1P–S1PR1 pathway has been clearly implicated in the regulation of angiogenesis, giving S1P a key role in regulating endothelial cell proliferation, migration, and tube formation (171). This was demonstrated by research that showed mice with a S1PR1 global knockout die from embryonic hemorrhage due to a lack of vascular maturation (172) and the recapitulation of this phenotype by a combined knockout of *SphK1* and *SphK2* (173). Also, the dominant role of extracellular S1P in angiogenesis was confirmed by its significant inhibition with neutralizing anti-S1P antibodies (12). The involvement of sphingolipids in MM-associated angiogenesis has led to efforts to study the therapeutic effect of different anti-angiogenesis approaches in myeloma.

Thalidomide, an immunomodulatory drug that can modify or regulate the immune system, is clinically recognized as an efficient therapeutic agent for MM. Beside its anti-inflammatory and anti-cancer activities, thalidomide has been thought to exert antiangiogenic action through an unknown mechanism. In a study established to uncover this mechanism, Yabu et al. showed that thalidomide inhibits cell growth and induces generation of ceramide through activation of neutral sphingomyelinase in human umbilical vein endothelial cells (HUVECs). Moreover, the treatment also depleted VEGF receptors on HUVECs. Exogenous S1P prevented thalidomide-induced growth inhibition and restored VEGF receptors to the control levels. The results of this study suggest that angiogenesis is regulated by the balance between ceramide and S1P-mediated signals which regulate expression of the VEGF receptors Flk-1 and neuropilin-1 (174).

These findings point out the importance of ceramide and S1P sphingolipids in MM-related angiogenesis. Although there has been a lot of progress in understanding MM-associated angiogenesis, the implication of sphingolipid metabolism in this aspect of MM development is still profoundly understudied.

MM cells can develop autocrine supportive loops and become independent from stromal sustenance, invade the extramedullary soft-tissue, and form extramedullary plasmacytomas (EMPs) (175), a condition difficult to treat and associated with a worse prognosis (176). Before developing into EMP and finally localizing in peripheral tissue, the metastatic plasma cells leaving the BM enter into the blood in the form of clonal circulating plasma cells (cPCs) (177). It is believed that *S1PR2* promotes confinement of B cells within the follicle center since loss of S1PR2 expression in *S1PR2-/-* mice is associated with loss of germinal center confinement, abnormal growth of B cells and development of B-cell lymphomas (178). A single-cell RNA-seq study comparing BM MM cells and peripheral cPC in five EMP-positive patients found *S1PR2* expression is downregulated in cPCs. The authors suggested that *S1PR2* activation negatively regulates migration and invasion of MM cells. By pharmacological and molecular loss of function studies, decreased *S1PR2* in U266 cells was reported to significantly promote MM cell migration and invasion through the upregulation of NF-κB phosphorylation status and increased MMP9 expression. Although this study didn’t provide a deeper mechanistic investigation, it suggests S1PR2 signaling negatively regulates EMP development and that its downregulation may partially account for the hematogenous myeloma cell dissemination to extramedullary sites (179).

## 5 Sphingolipids as Mediators of the Immune Response in MM Progression

The immune microenvironment plays a crucial role in tumor evolution and progression and contains different cell populations such as macrophages, natural killer cells (NKs), dendritic cells (DCs), and effector T cells that can play a role in the antitumor immune response. Their effect is counterbalanced by the tumor cells which induce an immunosuppressive microenvironment by promoting the development of cells such as myeloid-derived suppressor cells (MDSCs) and regulatory T cells, thus protecting themselves from destruction.

Malignant plasma cells from patients with newly diagnosed MM express high levels of the CD1d, a nonpolymorphic β_2_-microglobulin associated and glycolipid-presenting human leukocyte antigen class I-like molecule. The glycolipid antigens presented by CD1d are recognized by invariant Natural Killer T cells (iNKT) cells, a small population of regulatory T lymphocytes defined by co-expression of T and NK cells markers (180). There are 2 major subsets of NKT cells: type I or invariant NKT (iNKT) cells, lipid-reactive T cells restricted to lipid antigens presented by CD1d molecules that recognize the prototypic antigen α-galactosylceramide (α-GalCer), and type II or diverse NKT cells that do not recognize α-GalCer (181). Upon activation, iNKT cells secrete proinflammatory cytokines that lead to rapid downstream activation of several immune cells, including NK, dendritic and T cells and also mediate anti-myeloma effects by anti-angiogenesis (182, 183). Depending upon the mode of iNKT activation by the tumor or exogenously derived glycolipids and the cytokine microenvironment, iNKT cells can either suppress or promote antitumor immunity (183). During the evolution from the premalignant MGUS to active myeloma, MM cells suppress immune antitumoral responses by inducing a marked deficiency of ligand-dependent interferon-γ production by iNKT cells (184). Myeloma cells that express CD1d are sensitive to lysis by iNKT cells, but the CD1d expression is almost totally lost in the advanced stages of MM and in the myeloma cell lines (185, 186) as part of the immune evasion mechanisms of this cancer by contributing to iNKT cell anergy in MM (3). A characteristic of malignant transformation is represented by the alterations in the glycosphingolipids synthesis in the cell membrane and their glycosylation pattern, thus inducing resistance to chemotherapeutic agents and evasion from apoptosis (3). The role of altered glycosphingolipids expression in myeloma pathogenesis was suggested by studies of Gaucher disease showing that accumulation of heterotypic glycolipids leads to hematolymphopoietic hyperplasia (187). In a significant percent of MGUS cases, either Gaucher disease-related or sporadic, the driver antigens that result in the production of the pathologic paraprotein are lyso-glucosylsphingosine (LGL1) and lysophosphatidylcholine (LPC), thereby linking glycosphingolipids to myeloma development (90). These studies indicate that a better understanding of the nature of lipid ligands recognized by iNKT cells may provide novel insights into the mechanism of the observed iNKT dysfunction and, also provide a rationale for further clinical studies to boost or restore iNKT cell function in myeloma.

Studies of the tumor microenvironment showed that monocytes and macrophages have a significant role in cancer associated inflammation. TAMs have a fundamental role in MM pathogenesis by promoting BM plasma cell homing and proliferation, angiogenesis and also supporting MM immune evasion and progression (106, 188, 189). TAMs protect MM cells from therapy-induced apoptosis, promote immune-escape (190), and also participate in inducing resistance to commonly used anti-MM drugs such as melphalan and bortezomib (92, 191). Although less studied directly in the context of MM, S1P is one of the microenvironmental factors that can regulate the interplay between immune cells and cancer cells. There is a big gap in the research concerning the role of sphingolipids in mediating BM macrophage polarization in MM, however it is likely that many of the principles of S1P biology gleaned from the study of the tumor microenvironment in relationship with other cancers, may be applicable more broadly, particularly in the context of MM. Accumulating evidence indicates a critical role for S1P in macrophage polarization as demonstrated in several models of cancer where S1P was found to induce a M2 phenotype in macrophages (70). It has been demonstrated that mesenchymal stem cells and TAMs sustain MM cell survival and proliferation through IL-6 and IL-10 cytokine production (192). TAMs from SphK2-deficient tumors displayed a pronounced anti-tumor phenotype, showing an increased expression of the pro-inflammatory markers/mediators such as NO, TNF-α, IL-12 and MHCII and a low expression of anti-inflammatory IL-10 and CD206. Thus, SphK2 generated S1P enhances the supportive character of the tumor microenvironment by modulation of macrophage polarization towards the M2 phenotype (193). A better understanding of the S1P role in the regulation of macrophage production and polarization in MM could lead to the development of new therapies which may target TAMs by reprogramming them toward an M1 phenotype and activation of their antitumor response.

S1P has come into view as a central mediator in the trafficking of immune cells, including B and T lymphocytes, NK cells, neutrophils, dendritic cells, macrophages, hematopoietic progenitors and mast cells through activating S1PRs on these cells. Unlike the knowledge accumulated for T cells, whose thymus and lymph node egress is regulated by S1P-S1PR1, the sphingolipid metabolism and signaling requirements for B cell migration and maturation are less well understood, and there is little research regarding the trafficking of MM cells in the context of sphingolipid metabolism. Unlike for B lineage cells in the lymph nodes and spleen, studies show that S1P-S1PR1 signaling is only weakly influencing the egress of normal B lymphocytes from BM (194). The efficient transfer of newly generated immature B cells from the BM to the blood requires S1PR1 signaling (157) and S1PR3 appears to be required for normal development of B cells in BM (195). The roles of the other S1PR in B lineage cells are quite unclear. It will be of interest, and of potential clinical importance, to determine if a dependence on S1P-S1PR signaling extends to MM clones for the control of their emergence into the blood and their extramedullary trafficking.

## 6 Sphingolipids as Mediators of Drug-Resistance in MM

Sphingolipids have been reported to play a role in the development of drug resistance and radio-resistance in several solid tumor cancer types as well as in chronic lymphocytic leukemia (CLL) (196). This can arise both through altered sphingolipid metabolism within the cancer cells as well as from changes within microenvironmental cells that then secrete an altered bioactive lipid profile. In many instances, increased levels of enzymes that push sphingolipid metabolism away from ceramide, thus decreasing the ceramide levels, result in reduced apoptosis and increased anticancer drug resistance.

### 6.1 Transporters

In some cases, it’s the drugs themselves that alter gene expression of enzymes involved in sphingolipid metabolism. For instance, doxorubicin upregulates expression of GCS (encoded by the *UGCG* gene) (197), which catalyzes glucosylceramide from ceramide, the precursor for all complex glycosphingolipids, which then leads to drug resistance. Besides depleting the pro-apoptotic ceramide, the complex glycosphingolipids cluster into plasma and subcellular organelle membrane microdomains and can enhance signal transduction to promote cell proliferation and survival. Overexpression of the GCS enzyme has also been linked to increased expression of the ABCB1 lipid transporter protein (also called P-glycoprotein and encoded by the *MDR1* gene) in several cancer cell types, which leads to increased anticancer drug efflux and multidrug resistance (196). All these properties would increase resistance to multiple anti-cancer drugs *via* different pathways that converge on GCS. Overexpression of GCS could also arise from hypomethylation of the promoter or altered signaling as cancers progress and acquire drug resistance. Therefore, methods that down-regulate or inhibit GCS might be expected to re-sensitize cancer cells to therapeutic drugs. In myeloma patients and MM cell lines, treatments with classic chemotherapeutic drugs, such as doxorubicin and melphalan, have been reported to upregulate ABCB1 expression, which contributes to the development of resistance to these drugs (198). However, it’s unclear if the upregulation of ABCB1 in these cells results from increased GCS. Further, it was reported that the GCS inhibitor eliglustat did not induce ABCB1 protein on the cell surface in the KMS-18 MM cell line (199). Fu et al. (162) reported that in U266 MM cells, ABCB1 (mRNA and protein) was expressed and upregulated by addition of extracellular S1P. However, the S1PR inhibitor FTY720 decreased ABCB1 expression, thereby potentially decreasing the possibility for drug resistance even as it induced apoptosis and autophagy in the cells. The response of MM cells to immunomodulatory drugs such as thalidomide, lenalidomide, and pomalidomide do not appear to be much affected by changes in ABCB1, although pomalidomide may be a ABCB1 substrate (196). There is controversy about whether bortezomib is a ABCB1 substrate, but carfilzomib is, and therefore MM cells with increased ABCB1 are more carfilzomib resistant. However, in many MM cell lines, ABCB1 expression is very low on the cell surface, and the specific ABCB1 inhibitor tariquidar did not enhance the sensitivity to either bortezomib or carfilzomib, so the relevance to MM biology and proteasome inhibitor resistance is unclear (199). A few studies suggest that ABCG2 in MM cells may contribute to intrinsic drug resistance, which is increased when the cells are exposed to ABCG2 substrates such as doxorubicin or topotecan (200, 201).

### 6.2 Enzymes of the Sphingolipid Rheostat

Sphingomyelin can be generated from ceramide (**Figure 1**) by two Sphingomyelin synthase (SMS) isoforms (SMS1/2 encoded by *SGMS*1/2) (202). Sphingomyelin synthesis has recently been reported to be important in generating PI-resistance in MM cells (203). SMS inhibitor D609 had a larger cytotoxic effect in PI-resistant cells (bortezomib and carfilzomib) than in the parental sensitive cells, inferring that the PI-resistant cells had become dependent upon higher levels of sphingomyelin or on the potential concurrent decrease of ceramide. They also reported that D609 synergized with both PIs when used on primary MM cells from multi-drug refractory MM patients.

Acid SM (ASM), which resides in lipid rafts in the plasma membrane and in lysosomes, and is also secreted, not only acts as an SMase, but is also a form of phospholipase C (PLC) (reviewed in (204)). Furthermore, ASM can cleave a large number of different phosphoplipids besides sphingomyelin, including the cellular growth factor ceramide-1-phosphate (Cer1P). So, the balance between SMS and SMase activity may prove important as a resistance mechanism.

A recent study reported by Faict and colleagues found ceramide species (d18:1/16:0), (d18:1/18:0) and (d18:1/24:1 (15Z)) increased in peripheral blood plasma from MM patients versus healthy subjects while sphingomyelin (d18:1/22:0) was decreased (205) and both acid and neutral SMase gene expression was elevated in both primary MM cells and MM cell lines (JJN3, OPM2, LP1, U266). Of key interest, exosomes from OPM2 and U266 cells had much higher levels of ASM protein than exosomes from LP1 and JJN3 cells. U266 cells are relatively drug resistant and ASM-high U266 exosomes could transfer resistance to both melphalan and bortezomib to JJN3 cells, whereas ASM-low JJN3 or LP1 exosomes did not help. Treatment of donor or recipient cells with an ASM inhibitor (amitriptyline) blocked the transferred drug resistance. Further amitriptyline was able to variably help sensitize cells to melphalan and/or bortezomib across the 4 cell lines and primary MM cells. However, it’s not clear whether this is a result of the decreased sphingomyelin, the generation of a particular ceramide type, if ceramide is directly acting to increase viability, or the ceramide gets used to make other bioactive lipids that are anti-apoptotic, or if it results from ASM action on one of the many other targets it has. Ceramides with differing carbon chain lengths and fatty acid lengths often have diverse consequences on cell physiology. Besides ceramide being processed into sphingosine and S1P, it can also get converted into other molecules that are proliferative such as Cer1P by the ceramide kinase (CERK), which supports production of cytokines and chemokines (206), or get converted into glycosphingolipids and increase microdomain signaling.

Upregulation of the acid ceramidase ASAH1 has been linked to apoptosis resistance due to depletion of ceramide in several solid tumors, as well as AML, while the alkaline ceramidase ACER3 promotes AML and hepatocellular carcinoma and indirectly protects against colon cancer by tamping down the innate immune system (reviewed in (207)). None of the ceramidases have been studied in MM cells and if they have roles in stimulating resistance to current therapies is unknown. However, in a survey of sphingolipid enzyme mRNAs in CD138^+^ plasma cells from healthy subjects and MM patients reported by Wallington-Beddoe and colleagues, among the ceramidases, only ACER3 was elevated in MM (208).

Depletion of ceramide, as a consequence of SphK1 activation, can lead to inhibition of PP2A activity and stabilization of PP2A targets, such as BCR-ABL and c-Myc, whose phosphorylation induces ubiquitination and proteasomal degradation (36, 209). Thus, overexpression of SphK1 in chronic myelogenous leukemia (CML) can increase resistance to the tyrosine kinase inhibitor imatinib by enhancing the stability of its target, the mutated kinase BCR-ABL. In MM cells, we showed that elevated transcriptional repressor GFI1, conferred enhanced MM cell viability and growth, and also increased resistance to bortezomib-induced cell death (163). We recently showed that the mechanism by which GFI1 enhanced MM viability in MM cells independently of p53 status is by repression of the *SGPP1* gene and protein, and indirect promotion of activated SphK1 protein, together resulting in increased intracellular S1P levels in the MM cells (36). Although we have not studied the GFI1-dependent bortezomib-resistance mechanism in MM, a possible role for intracellular S1P emerged. This possibility is also supported by mouse models of breast and prostate tumors where *SGPP1* silencing mediated by miR-95 generated S1P-dependent radiation resistance (210).

SphK2, but not SphK1, expression was reported to be increased in the primary MM cells from patients versus healthy subjects and in MM cell lines (164, 208). While elevated SphK2 has contrasting roles in cancers of different types (211), as discussed in detail below in section 7, inhibition of SphK2 synergizes with PI drugs to kill MM cells and can re-sensitize resistance MM cells (208, 212). However, the question as to whether elevated SphK2 causes PI resistance, or resistance to other therapeutics, in MM has not been directly addressed.

Besides consideration of S1P production within the MM cells, extracellular S1P produced by cells in the microenvironment, such as BMSC or mature osteoclasts in response to the presence of the MM cells, can increase the drug resistance of the MM cells. S1P serum levels have been reported to be higher in patients with MM, even those with anemia, than in healthy subjects (213). Tanaka et al. (213) also showed that exogenous S1P interfered with the efficacy of MM responses to PIs, suggesting that S1P-S1PR signaling regulates the proteasome. Thus, microenvironmental sources of extracellular S1P can contribute to the drug-resistance of MM cells and provide another reason why anti-S1P metabolism therapeutics may be useful and argue the importance of testing these *in vivo*, particularly for re-sensitization to other therapeutics. Overall, a better understanding of the roles that the S1P metabolic pathways may play in therapeutic resistance would likely provide the possibility of additional therapeutic targets to re-sensitize drug-resistant MM.

## 7 Therapeutic Considerations for Sphingolipids in MM

Cancer cells, in general, are characterized by the constitutive activation of several pro-survival pathways combined with overexpression of anti-apoptotic protein family members. Moreover, cancer cells remodel their microenvironment so that its components add another layer of support for their growth. MM arises from a malignant immune cell that is developing in the immune microenvironment of the BM, which plays a crucial role in tumor evolution and progression. Therefore, it is unlikely that single agent therapy will be sufficient for such a complex disorder. Accumulating evidence suggest that, because of their importance in cell signaling that ultimately determines cell fate, involvement in the resistance to current chemotherapeutic options, importance in immune processes and bone remodeling, bioactive sphingolipids have a great potential to be targets in anti-myeloma therapy, especially in effective combinations with other drugs. Therefore, the deregulation of enzymes that control S1P synthesis and catalysis may provide prospects for therapeutic intervention to indirectly influence the receptor-mediated or intracellular target-mediated effects of S1P. A few studies have exploited the key role of the sphingolipid metabolism components for their therapeutic potential in MM. Generally, these approaches can be categorized into interventions that induce MM cell death by indirectly engaging sphingolipid pathways and treatments that address sphingolipids as direct targets.

### 7.1 Sphingolipids as Mediators of Cell Death Signaling

New cytotoxic therapies are focused on designing small molecule inhibitors that interfere with the pro-survival function of oncoproteins such as anti-apoptotic BCL2-like proteins. One of the most promising such molecules, ABT-263/737, is a small molecule BH3 mimetic with a very high specificity for BCL2, BCLxL and BCLw, and has shown promise against many tumor types, as a single agent and in combination therapeutic strategies (214). ABT-263 inhibits BCL2-like anti-apoptotic molecules by binding to their hydrophobic pocket and displacing the bound pro-apoptotic BCL2-like proteins, including BAX and BAK, to induce their activation followed by mitochondria outer membranes permeabilization (MOMP). Beverly et al. showed that in the RPMI8226 myeloma cell line, ABT-263, release of BAK allowed it to activate mitochondrial ceramide synthase and induced generation of long-chain ceramide C16. Ceramide C16 then induced BAX/BAK aggregates, along with synergistic channel formation by ceramide and BAX/BAK into the mitochondrial outer membrane (215). The study pointed out that due to its low affinity for the hydrophobic pocket of MCL1, ABT-263 is unable to inhibit the MCL-1 anti-apoptotic functions. As a consequence, overexpression of MCL-1 in RPMI8226 cells prevented the ability of this drug to induce ceramide generation, which presents elevated MCL-1 as a mechanism of developing resistance to this drug.

While investigating the therapeutic value of Fenretinide (N-(4-hydroxyphenyl) retinamide, or 4HPR), a neoclassical analog of all-trans retinoid acid (ATRA) in MM, Li et al. showed its ability to dose-dependently inhibit cell growth and induce caspase-dependent apoptosis in MM cell lines. The authors suggest that this 4HPR effect is mediated by ceramide accumulation based on the counteractive effect of exogenously added S1P (mimicking the microenvironmental conditions) (216). Although ceramides were previously reported as major mediators of 4HPR action in other tumor types (217, 218), this fact is still insufficiently demonstrated in MM where molecular alterations and functional enzymatic studies are needed to validate this observation.

### 7.2 Sphingolipid Metabolism Components as Direct Targets in MM Therapeutical Approaches

#### 7.2.1 S1PRs as Therapeutic Targets

Extracellular S1P controls several cellular processes, including growth and survival, acting in both autocrine and paracrine ways and by interacting with different S1P receptor subtypes. Thus, S1PR are attractive therapeutic targets due to their wide distribution and functional diversity. There are only a few studies exploring the effect of S1PR targeting in MM pathogenesis and they all investigate the role of FTY720 (also known as Fingolimod/Gilenya) as a potential anti-MM therapeutic strategy. FTY720 is a structural analogue of sphingosine derived from myriocin (ISP-1), a metabolite of the fungus *Isaria sinclairii*, which acts as an agonist of four out of the five S1PRs (S1PR1 and S1PR3-5) (219).

Yasui et al. showed that FTY720 significantly dose- and time-dependently inhibited growth of several drug-sensitive and drug (dexamethasone and doxorubicin)-resistant MM cell lines, as well as tumor cells from two patients with relapsed MM refractory to conventional therapies. In contrast, FTY720 had only a small effect on normal cells (peripheral blood mononuclear cells and BM mononuclear cells). The authors showed that FTY720 induces MM cell death *via* caspase 8, -3 and -9 activation, alterations in mitochondrial membrane potential, release of mitochondria proteins (cytochrome c and Smac/Diablo), cleavage of proapoptotic BAX protein and PARP, all of which define apoptotic cell death. Moreover, the study demonstrated that FTY720 treatment was effective even in the presence of the pro-survival cytokines IL-6 and IGF-1, or adherence of MM cells to BM stromal cells, suggesting this dug can overcome the protective BM milieu (220). Furthermore, FTY720 enhanced the cytotoxicity in preclinical models by dexamethasone, which induces intrinsic apoptotic signaling, and anti-Fas antibody, which induces extrinsic apoptotic signaling, supporting the rational for combination therapy of many drugs with FTY720 for MM (220).

In a later study, Liao et al. confirmed the cytotoxic and pro-apoptotic, caspase-3 dependent effect of FTY720 on MM cells using the U266 cell line. These studies showed that FTY720-induced autophagy also contributed to the cell death and promoted apoptosis, implying crosstalk. Both processes relied upon FTY720 generation of reactive oxygen species (ROS) (221). Based on the conclusion that FTY720-induced cell death could not be fully explained by these two mechanisms, the authors followed up with another study depicting the role of ferroptosis in FTY720-induced MM cell death. They also suggested another mechanistic aspect of FTY720 function by activation of PP2A followed by AMPK dephosphorylation and eEF2 ending with increased autophagy and ferroptosis, which then reinforce each other through a positive feedback loop that promotes MM cell death (222).

Beider et al. report reaffirmed the FTY720 effect on MM cells viability, and additionally identified CXCR4 as a molecular target of FTY720, thus suggesting a functional crosstalk between the CXCR4 and S1P pathways in MM. These authors described an activating interaction between S1P and CXCR4/CXCL12 signaling pathways important for MM cell survival (223). This finding is important because the authors observed an upregulation of CXCR4 expression in MM cells with increasing bortezomib treatment, and as a consequence, CXCR4/CXCL12 expression decreased the responsiveness of the MM cells to bortezomib. Of importance, FTY720 effectively killed bortezomib-resistant cells, and the combination of FTY20 with bortezomib resulted in increased cell death displaying elevated pH2AX in both bortezomib-sensitive and -resistant MM cells. Thus, suggesting that the mechanism of combinational treatment involves the induction of DNA damage and that FTY720 may be a potent apoptosis-inducer that acts independently of the proteasome pathway. The effect of FTY720 was validated in an *in vivo* model of CXCR4-driven human MM engraftment in murine BM where it significantly reduced the tumor load in the BM niche (223).

FTY720 was studied also in combination with other anti-MM agents, such as metformin. Zhao et al. showed recently that this combination synergistically inhibited viability and induced caspase 3-dependent apoptosis in MM cells. The authors showed that the metformin and FTY720 combo induced ER stress and inhibited the PI3K/Akt/mTOR pathway through ROS generation in MM cells (224).

Although all these studies discuss FTY720 as a prodrug that is a S1PRs agonist, none of them acknowledge that the antiproliferative effects of FTY720 are mediated independently of S1PRs. The S1PR-dependent effects of this drug are elicited following its phosphorylation by intracellular SphKs. FTY720 is phosphorylated by SphK2 to form phospho-FTY720 and only with low efficiency by SphK1. Phospho-FTY720 is exported extracellularly *via* the SPNS2 transporter, and acts as a functional antagonist for S1PRs, thereby exhibiting its immunosuppressive effects (225). The role of FTY720 in its complexity, as a direct anti-cancer cell and immunosuppressant, remain elusive in MM. More exhaustive studies are needed to address the impact of S1PRs antagonists on the microenvironmental components, including the immune response, besides the revealed direct anti-myeloma effect.

#### 7.2.2 Lipid-Raft Targeted Therapy

Membrane fluidity is one of the most important physiochemical properties of the cell membranes and plays an essential role in the regulation of cell viability (226). Plasma membrane specific organization involves nanoscale membrane microdomains known as lipid rafts that regulate several cellular processes including intracellular signaling, redox balance and cell death (227, 228). Therefore, alterations in the composition and organization of these microdomains affects cellular functions, signal transduction, membrane plasticity, and membrane trafficking (229). As a hallmark of their adaptation to the microenvironment, cancer cells reorganize their plasma membranes to preserve proliferation, escape apoptosis and resist anticancer drug treatments (230). A critical problem of the anticancer therapy in general, and of MM in particular, is the development of multidrug resistance (MDR), which represents a decreased free diffusion of anticancer drugs through the plasma membrane or enhanced facilitated export (231). Moreover, MDR increases sphingomyelin synthesis, thereby keeping a low ceramide level, which implies a decrease in ceramide-enriched lipid rafts are involved in the induction of cell death (232).

In contrast, activation of ASM increases membrane fluidity through ceramide generation from sphingomyelin and amplifies death receptor signaling by inducing lipid-raft clustering (233). A recent study by Tsukamoto et al. showed that expression of ASM is abnormally elevated in myeloma cell lines relative to normal PBMCs (234). Furthermore, the authors show that EGCG ((−)-epigallocatechin-3-O-gallate), the major polyphenol of green tea, activates the PKCδ/ASM pathway through the 67LR (67 kDa laminin receptor) in MM cells, whose expression is abnormally high in these cells. PKCδ activation induces translocation of ASM to the plasma membrane, where it enhances apoptosis (234).

#### 7.2.3 Sphingolipid Enzymes as Potential Therapeutic Targets

The malignant plasma cells are required to sustain a high production of monoclonal immunoglobulin that involves folding in the endoplasmic reticulum (ER) lumen producing a significant amount of stress on the ER chaperones, which, as a consequence, activates the Unfolded Protein Response (UPR). The UPR is a homeostatic process aiming to correct the high ER stress levels by globally slowing protein translation and increasing expression of ER chaperones. The 26S proteasome counterbalances ER stress by degrading proteins that cannot be correctly folded by the ER, therefore its inhibition became a highly potent anti-myeloma therapeutical intervention. Both classes of 26S proteasome inhibitors, reversible (Bortezomib) and irreversible (Carfilzomib), have significantly improved the remission rates. However, disease relapses inevitably as proteasome inhibitor resistance develops. Therefore, ways are needed to re-sensitize patients to these highly effective agents. Recently, Wallington-Beddoe et al. found that inhibition of the ER membrane resident enzyme SphK2 is associated with the accumulation of proapoptotic ceramide and other upstream sphingolipids that IRE1 and ATF6 are directly sensitive to independent of the unfolded protein levels. Thus, this causes a marked increase in ER stress with a different pattern of UPR activation from that observed with proteasome inhibitors, specifically no significant increase in the lumenally-located ER chaperone BiP, although still activating the PERK-eIF2α and IRE1-XBP1 pathways. The different mechanisms leading to ER stress induced by bortezomib and SphK2 inhibition provided the rationale for concomitantly targeting both pathways in myeloma demonstrating a synergistic anti-MM effect *in vitro* and *in vivo*. Further, inhibition of SphK2 is able to re-sensitize PI-resistant cells regardless of the PI or the mechanism of resistance within the proteasome (208, 212).

Venkata and collaborators recently showed that SphK2 represents a potential therapeutic target for the treatment of MM by using ABC294640, a SphK2-specific inhibitor that exhibited anti-myeloma activity in primary human CD138^+^ cells and in *in vivo* mouse xenograft models. ABC294640 (235–237) has been tested in a single-agent phase I clinical trial in patients with advanced solid tumors demonstrating good pharmacokinetics, oral bioavailability, and biodistribution. Additionally, these authors demonstrated that, besides its activity when applied alone, ABC294640 acts synergistically with the Bcl-2 inhibitor (ABT-737) in killing myeloma cells. This finding may be particularly important in the treatment of relapsed or refractory MM patients, who become resistant to currently available agents such as dexamethasone, immunomodulatory drugs, and proteasome inhibitors (164).

Activation of neutral sphingomyelinase by thalidomide induces a transient increase of ceramide content. This increase in ceramide has been shown to mediate the antiangiogenic effect of thalidomide in model using human umbilical vein endothelial cells (HUVECs) (174). Although thalidomide is recognized as an efficient therapeutic agent for multiple myeloma, there are no studies yet to address its mechanism of action on angiogenesis in this pathology.

#### 7.2.4 Sphingolipid Species as Therapeutic Agents

During competition for survival, myeloma suppress the hosts’ immune system to evade antitumor immunity. Consequently, cytotoxic T cells will have reduced proliferation and expansion and NK and iNKT cells will enter an anergic state. As already mentioned, the CD1d expressing MM cells are sensitive to lysis by interferon-γ secreting iNKT cells but clinical progression in MM patients is associated with fewer activated iNKT cells due to depletion of MM CD1d expression (184). This functional defect could be overcome using dendritic cells pulsed with the iNKT ligand, α-GalCer, which binds tightly to CD1d. When α-GalCer ligand was injected alone in humans with cancer, no substantial iNKT expansion was observed. However, injection of glycolipid loaded dendritic cells led to prolonged NKT activation in mice. These results led to approaches that utilize targeted antigen presenting cells to enhance NKT cell activation (238). Injected α-GalCer loaded mature human dendritic cells activate and increase circulating iNKT cells *in vivo* but were still defective in activating downstream innate immune functions (239). Richter et al. showed that combining α-GalCer loaded mature monocyte-derived dendritic cells with immunomodulatory drugs such as lenalidomide, thereby providing antigen-dependent co-stimulation of both NKT cells and T cells, led to synergistic activation of innate lymphocytes *in vivo* and mediated antitumor effects in asymptomatic myeloma patients (240). Nur and coworkers used a 5T33MM myeloma mouse model to also investigate the therapeutic effects of α-GalCer. Treatment with α-GalCer during the course of the disease showed a clear reduction in tumor load and paraprotein, which was also associated with a reduction in microvasculature density, even in mice with a higher tumor burden. These results indicate that α-GalCer has both direct anti-angiogenic effects and anti-tumor effects (241).

This knowledge reveals a complex landscape and a possible role for several immune cells that may participate in the immune-mediated tumor growth control and, also suggests that combination approaches may be necessary for immune-based prevention or therapy of MM.

## 8 Conclusion and Future Perspectives

Increasing experimental proof supports the importance of sphingolipid metabolism in MM progression. New insights into the bioactive sphingolipid pathways were enabled due to advances in important technological developments and highly quantitative analytical methodology. Although until recently, most efforts to identify novel targets have focused on molecular pathways at genomic and transcriptomic levels, metabolomic studies are becoming more prevalent. It is widely accepted that evaluation of metabolomic reprogramming in cancer cells provides essential information about tumorigenesis, however, studies that focus on the metabolic changes occurring during MM development and MM relapse are only in their emergent stages. The recent discoveries of the underlying genetic and epigenetic alterations in MM are hindered by the high molecular diversity and complexity of the clones, the variable response to therapy, and the appearance of chemoresistant clones to a variety of therapies. Therefore, integration of this knowledge with sphingolipidomic data may provide not only information about the MM cells per se, but an insight into how myeloma interacts with, and exploits, existing BM niches. This will improve our understanding of how to treat relapses and advance anticancer efficacy of immunotherapy. Nevertheless, further detailed scientific investigation is required for a better understanding of which microenvironmental factors modulate alterations of the key enzymes and the mechanism underneath these alterations that change the intracellular sphingolipid metabolite composition and leads to MM progression. This new knowledge may help identify novel prognostic markers to assess the risk of MM development from MGUS, and new targets for therapeutical approaches. Since increased sphingolipid metabolism towards glucosylceramide and/or S1P generation results in resistance to anticancer therapy, they contribute to the importance of finding therapeutic strategies to treat MM relapses and evade the acquired drug resistance. Moreover, therapies that combine the ability to kill MM cells and regain bone mass will be the most desired innovative strategies.

## Author Contributions

DP: conception and design; DP and DG: manuscript writing; DP, KL, and DG: manuscript editing; KL: funded the publication. All authors have read and agreed to the published version of the manuscript.

## Funding

This work was supported by the ACS-IRG-16-192-31 (DP), NIH/NCI R01CA121044 (KL) and NIH/NIAMS R01AR059679 (DG).

## Conflict of Interest

The authors declare that the research was conducted in the absence of any commercial or financial relationships that could be construed as a potential conflict of interest.

## Publisher’s Note

All claims expressed in this article are solely those of the authors and do not necessarily represent those of their affiliated organizations, or those of the publisher, the editors and the reviewers. Any product that may be evaluated in this article, or claim that may be made by its manufacturer, is not guaranteed or endorsed by the publisher.
